# Antimicrobial Resistance Gene Detection Methods for Bacteria in Animal-Based Foods: A Brief Review of Highlights and Advantages

**DOI:** 10.3390/microorganisms9050923

**Published:** 2021-04-26

**Authors:** Beatriz S. P. Galhano, Rafaela G. Ferrari, Pedro Panzenhagen, Ana Carolina S. de Jesus, Carlos A. Conte-Junior

**Affiliations:** 1 Molecular & Analytical Laboratory Center, Faculty of Veterinary, Department of Food Technology, Universidade Federal Fluminense, Niterói 24220-900, Brazil; b.galhano1@gmail.com (B.S.P.G.); pedronepl@yahoo.com.br (P.P.); conte@iq.ufrj.br (C.A.C.-J.); 2 Food Science Program, Chemistry Institute, Universidade Federal do Rio de Janeiro, Rio de Janeiro 21941-901, Brazil; 3 Center for Food Analysis (NAL-LADETEC), Universidade Federal do Rio de Janeiro, Rio de Janeiro 21941-901, Brazil; carolana08@outlook.com; 4 Agrarian Sciences Center, Department of Zootechnics, Federal University of Paraiba, Paraiba 58397-000, Brazil; 5 National Institute of Health Quality Control, Fundação Oswaldo Cruz, Rio de Janeiro 21040-360, Brazil

**Keywords:** antibiotic resistance transmission, meat chain, microbiology molecular tools, growth promoters

## Abstract

Antimicrobial resistance is a major public health problem and is mainly due to the indiscriminate use of antimicrobials in human and veterinary medicine. The consumption of animal-based foods can contribute to the transfer of these genes between animal and human bacteria. Resistant and multi-resistant bacteria such as *Salmonella* spp. and *Campylobacter* spp. have been detected both in animal-based foods and in production environments such as farms, industries and slaughterhouses. This review aims to compile the techniques for detecting antimicrobial resistance using traditional and molecular methods, highlighting their advantages and disadvantages as well as the effectiveness and confidence of their results.

## 1. Introduction

Antimicrobial agents are widely used in animal production as prophylactics and therapeutics and in small doses as growth promoters [1,2,3]. These drugs are also used in human medicine, and their misuse or overuse in both human and veterinary medicine can select resistant bacterial strains [4,5,6]. The use of antimicrobials as growth promoters began in the 1950s with sub-therapeutic doses in animal diets, allowing a better ratio between ingested feed and weight gain [1]. Seeking those benefits over the years brought a neglected consequence: the emergence of resistant microorganisms. Consequently, in 1969, in the UK it was determined that antimicrobials used in human medicine with therapeutic purposes could only be used as growth promoters if therapeutic use was limited and did not represent a risk of inducing cross-resistance to those used to treat human diseases [7].

In the middle of 1975, the first experiments were done showing the emergence of strains resistant to certain antimicrobials used in animal production [1]. At the same time, the European market banned the use of tetracycline as a growth promoter, and a decrease in resistance was observed for this drug in both animal production and human medicine, respectively. Over the years, some European groups (i.e., European Common Market and European Union) have banned other drugs formerly used as growth promoters, such as avoparcin [1,7]. Nevertheless, several bacterial strains have displayed resistance to a wide range of antibiotics used in veterinary medicine, such as the aminoglycosides, since 1980. Since 1984, a wide range of antibiotic resistance has been observed in strains of *Escherichia coli*, and later in other bacteria such as *Salmonella* Typhimurium and *Klebsiella pneumoniae* [7,8,9]. These microorganisms show plasmid-mediated gene transfer as a resistance mechanism and the capacity to quickly colonize both animals and humans [4,10,11]. Overall, bacteria develop resistance by various mechanisms that may be intrinsic or acquired through mutations and horizontal gene transfer. The latter can occur by direct contact through the conjugation of resistant bacteria and susceptible bacteria, by transformation through the acquisition of free DNA in the microenvironment, and through the transfer of DNA mediated by bacteriophages, a process called transduction [9,12,13].

Humans can be exposed to resistant bacteria of nosocomial and/or community origin. In nosocomial infections, antimicrobial resistance threatens the success of controlling the bacteria and causes admission to the intensive care unit (ICU), pneumonia and bloodstream infections [7,14,15,16]. Highly resistant bacteria such as MRSA or multidrug-resistant Gram-negative bacteria are the cause of high incidence rates of nosocomial infections worldwide [16,17,18]. In community infections, exposure may occur through direct contact with animals and humans or through ingestion of animal-based foods (ABF). Veterinarians, farmworkers and slaughterers have a high risk of becoming infected with resistant strains due to direct contact with production animals. However, consumers, even without direct contact, can acquire these infections through consumption of ABF [3,19,20], which in turn can lead to a larger number of hospitalizations, ineffective treatments and an increase in the number of deaths in consequence of infections by resistant bacteria [14,19,21,22]. The most effective way of preventing resistant strains from arising in ABF is to control the excessive use of antimicrobials in animal production, especially prohibited drugs, thereby avoiding treatment failures [17,23,24].

In this context, there are several methods of identifying resistant bacteria (phenotypic resistance) and their respective resistance genes (genotypic resistance). The traditional methods are primarily based on the culture of these microorganisms under specific conditions. Although simple and easy to carry out, some aspects are not so advantageous. The existence of viable non-cultivable microorganisms, or the long time that certain microorganisms may have to multiply in the environment, ends up being an obstacle to some researches [14,25,26,27]. On the other hand, molecular methods are essentially based on the amplification of target genes (i.e., PCR, real-time PCR (qPCR), multiplex PCR, random amplified polymorphic DNA (RAPD), PCR combined with restriction fragment length polymorphism (PCR–RFLP)), whole-genome sequencing and metagenomics [28,29,30]. Although more expensive than traditional cultivation, they are essential tools for the study of multiple microbiomes. Some of these techniques have great advantages, such as the fast execution time of qPCR and the identification of all resistomes in a sample using metagenomics. Each technique also has particular limitations, e.g., execution time, low reproducibility of results, instability of the RNA molecule, specific equipment, technicians capable of executing it, and bioinformatics expertise, most of them required for greater reliability of results [28,31,32]

This brief review aims to compile the techniques for detection of antimicrobial resistance (AMR) using traditional and molecular methods, highlighting their advantages and disadvantages as well as the effectiveness and confidence of their results in addressing some of the technical aspects, and then to discuss the application of these techniques for specific purposes.

## 2. Traditional Methods

The focus of using traditional methods for the detection of AMR is the identification and quantification of bacteria [28]. They are based on the morphological and biochemical characteristics of the colonies, allowing easy counting of the cultivable bacteria present in the medium [2,4]. They are considered standard techniques for identifying specific microorganisms, mainly due to their high sensitivity [14,15]. The basis of such methods is the screening of various bacterial cultures present in an environment of interest in a non-selective medium. After that, more selective media prevent the growth of certain cultures while favoring the permanence of the microorganism cultures of interest. Therefore, phenotypic analyses, such as microscopy techniques, enzymatic characterization and antibiotic susceptibility tests (e.g., minimum inhibitory concentration (MIC) in broth or agar, disk diffusion and E-test^®^), can be performed with the objective of characterizing lineage [23,33,34]. MIC is also a precursor technique to the Fractional Inhibitory Concentration test (FIC), used to evaluate combined therapies and the existing synergy between chemotherapeutic agents. The FIC is determined for each drug by dividing the MIC of each drug, when used in combination, by the MIC of each drug when used alone. The following formula is applied: FIC1 + FIC2 = FIC Index, where FIC1 is equal to the MIC of drug one combined, divided by the MIC of drug one alone. FIC2 is equal to the MIC of drug 2, in combination, divided by the MIC of drug two alone. The FIC index value is then used to determine whether synergism, indifference or antagonism has occurred between antibacterial agents. The FIC index lower or higher than 1 indicates synergy or antagonism, respectively, because fewer or more drugs would be needed to produce the same effect as drugs alone [35,36]. The technique is gaining prominence nowadays since the use of drugs/substances in combinations is increasingly necessary to improve response to the growing phenomenon of antibiotic resistance. Nevertheless, there are limitations associated with performing synergy tests, for example, in a typical microbiology laboratory, especially as there is a lack of accepted standards for tests such as FIC. Furthermore, the testing process is laborious, time-consuming and requires experience with specific procedures [35,36].

Although considered the gold standard for the identification of specific microorganism lineages, some authors portray their disadvantages. A remarkable downside is the time required for cultivation, as well as the inability to detect some microbial species if they are present in a reduced number in the medium [37]. Another drawback is viable but non-cultivable bacteria which, while maintaining their metabolic activity, cannot be cultivated in routine culture media. This happens due to some stress such as temperature, substrates or oxygen at concentrations inadequate for their growth. *Enterococcus faecalis*, for example, may have proteins and enzymes (e.g., GroEL and DnaK (general stress proteins) and ATP-synthase β-chain (enzymes responsible for ATP delivery) that enter in a non-cultivable state and which are expressed at lower levels compared to exponentially growing cells [38]. Additionally, qualified workers are required to execute such techniques since their elaboration needs a great number of stages and is considered an intensive labor [35]. Ambiguous phenotypic characteristics (e.g., the occurrence of *Campylobacter jejuni* with a negative result for hippurate (herbivore urea), are common misinterpretations considering the high technique specificity [39].

The most commonly used technique for identifying antimicrobial susceptibility is determination of MIC. This method has the purpose of quantifying the minimum concentration of antimicrobial that inhibits the apparent growth of bacteria when carried out in agar or broth. Solutions with a defined number of bacteria (generally 0.5 in the MacFarland standard) are inoculated on agar or broth which has a diluted antimicrobial concentration. At the end of the incubation period, it is observed if there is microbial growth. This is a simple low-cost method that does not require specialized equipment. Once the MIC is determined, the therapeutic concentration of an antimicrobial can be adjusted for effective treatment. On the other hand, it is not possible to determine resistance in viable non-cultivable bacteria, and success depends on the incubation time and diluted antimicrobial concentration as well as the number of inoculated bacteria. Overall, it is a semiquantitative technique, which may not determine an exact MIC value [24,40].

The disk diffusion method consists of the diffusion over agar by an antimicrobial impregnated in a paper disc, where inhibition of the microbial growth circle will occur around the disc. It is a qualitative method that classifies a sample as resistant, intermediate, or susceptible. Additionally, it is a practical and easy to carry out method, ideal for fast-growing bacteria. However, there are some limitations, e.g., the use of antimicrobials that do not diffuse well in agar, and difficult interpretation for fastidious and anaerobic microorganisms [23,41]

The E-test^®^ is a combination of the last two mentioned methods. It has disk diffusion-like processing; however, it determines MIC. A rectangular gadget is placed on an agar plate; on one side of this gadget is the antimicrobial concentration gradient and on the other is the interpretation scale. Even though the E-test^®^ possesses the limitations of the two tests previously mentioned (i.e., execution time), it has an immobilized antimicrobial gradient indicated on the ruler and it guarantees a simpler way of directly quantifying the susceptibility of microorganisms, especially those which are difficult to culture (e.g., *Haemophilus influenzae* and *Mycobacterium bovis*) or even anaerobes [23,34]

The cultivation of bacteria originating from ABF, especially from the meat production chain (e.g., surface swabs from both food and the production environment) has been widely performed around the worl [10,11,18,42]. Such studies have shown the constant presence of several microorganisms that are resistant and often multi-resistant to antimicrobials. Bacteria commonly found in this ABF are *Enterococcus faecium*, *Enterococcus faecalis*, *Escherichia coli*, *Listeria monocytogenes*, *Staphylococcus aureus*, *Vibrio parahaemolyticus*, *Yersinia enterocolitica*, *Bacillus cereus*, *Salmonella* spp. and *Campylobacter* spp. Authors have demonstrated resistance to several classes of antimicrobials such as tetracycline, ciprofloxacin, chloramphenicol, enteromycin, benzalkonium chloride, cadmium chloride, methicillin, streptomycin, ampicillin, gentamicin, nalidixic acid, sulfafurazole, vancomycin, clindamycin, amoxicillin, sulfonamides and ciprofloxacin [10,11,18,42].

Regardless of the advantages or disadvantages of traditional methods for detecting AMR in bacteria, they are essentially helpful for making the right choice for the most effective treatment of resistant bacteria in clinical infections. Nowadays, the scientific and medical community has already agreed that antimicrobial use prophylactically, or even as growth promoters, is the real cause of the increased resistance to many antimicrobial classes. The conscious use of these medicinal products must be respected both for productive and prophylactic purposes and for the treatment of production animals, respecting the dose and correct frequency of administration of these medicinal products. Such care helps to avoid interference in the treatment of human infections, as well as to reduce the resistant species, often multi-resistant to several antimicrobial drug classes.

Despite some disadvantages reported for traditional methods, they are still the most used to identify AMR due to their simplicity (Table 1). Even studies that use molecular methods to achieve their goal use traditional methods first due to their simplicity of performance, in addition to providing a general overview of the presence of the microorganisms of interest and AMR.

## 3. Molecular Methods

### 3.1. Polymerase Chain Reaction—PCR

PCR is an in vitro method that allows the exponential amplification of specific sequences of DNA and RNA, a technique applied widely due to its high specificity. PCR is used in the laboratory routine as a fast method of identifying bacteria from multiple environments, as well as resistance genes [15]. One of its significant advantages, when compared to traditional cultivation, is related to the possibility of amplification of genes from existing microorganisms that are not cultivable and/or are dead, and therefore not capable of being identified by traditional methods. Traditional methods often generate erroneous (e.g., false negative) interpretations based only on phenotypic characteristics, avoided when using conventional PCR techniques as a complement to the findings obtained by traditional techniques [43].

PCR can be optimized by a multiplex reaction, an improved method of the conventional PCR, as well as qPCR. In this method, several primers are used in the solution mix so that it is possible to identify and differentiate more than one type of microorganism in a single run. It is possible to analyze up to nine different DNA targets, which is a great choice for investigation of ABF bacteria due to the ability to detect many microorganisms at the same time. The great advantage is the reduced cost and time by amplifying different genes at the same time [44]. For example, Xu et al. [45] described multiplex PCR as a powerful tool for the correct, efficient and fast detection of *Vibrio cholerae*, *V. parahaemolyticus*, *V. vulnificus* and *V. alginolyticus* in seafood samples in a single analysis. On the other hand, all primers need to have a similar annealing temperature and their amplicons need to be marked differently from each other to prevent the interaction of various primers and eventual disruption of the amplification process [46,47]. These difficulties require extensive and laborious validation methods prior to analysis using multiplex PCR.

PCR has already proved to be suitable even for detecting the presence of point mutations associated with broad-spectrum AMR genes if one or both primers are designed to anneal at sites of sequence variation [27]. A study on South Korean farms showed the transfer of resistance genes to oxazolidinones and phenicol in 5 out of 10 *Enterococcus* isolates with resistance to linezolid by the detection of the transferable resistance plasmidial genes *optrA* and *fexA*. These results were a classic demonstration of the hazard to human health caused by multiple resistance transfer of last-resort antibiotic resistance genes among bacteria [48]. PCR was also used to detect antimicrobial multiple resistance genes isolated from *Staphylococcus aureus* obtained from milk, meat and other animal product samples from supermarkets. The results showed 19 out of 125 *S. aureus* isolates displaying multiple resistance to penicillin, enteromycin, kanamycin and tetracycline through identification of the genes *blaZ*, *msrA*, *ermB*, *ermC* and *tetK* [49]. Seo and Lee [11] also used the PCR technique to quickly investigate the presence of plasmid-mediated quinolone resistance genes in β-lactamase-producing *E. coli* from samples from laying hens. They identified 86 out of 142 *E. coli* isolates with multiple resistance to antimicrobials. Fifteen of these were β-lactamase producers and six had quinolone resistance genes (e.g., *qnrS1* and *qnrB4*). Additionally, Pishnian et al. [44] investigated the presence of resistance genes to colistin in 944 broiler cloacal samples. In their results, samples resistant to colistin were identified by PCR detection of the *mgrB* gene, which is responsible for the negative feedback regulation of signaling systems in several enterobacteria. Finally, PCR has shown that fishmeal can also be an important reservoir for the dissemination of resistance genes. A study conducted by Han et al. detected severe multiple resistance genes against 24 antimicrobial drugs in bacteria from fishmeal samples. The genes give resistance against five classes of important antibiotics for human medicine, with fluoroquinolone (*qnrA*) the most commonly found [50].

As we reported above, some successful studies have demonstrated the efficient and rapid use of PCR to detect AMR in bacteria. However, the success of PCR performance still depends on intrinsic and extrinsic factors such as the conditions in which the raw material is found (salting, freezing, pasteurization, ionizing radiation, etc.), cycle conditions, temperature, primer concentration and even the DNA extraction solution. In addition, the components of food matrices and the intrinsic properties of foods can interfere with tests for detecting pathogens and their genes. Studies have shown that food matrices, such as ground beef and chicken, can inhibit PCR assays [51]. One way to avoid such limitations would be through effective sample processing, as well as purifying and/or concentrating the target pathogen before nucleic acid amplification [52]. Finally, the conventional PCR technique can detect gene sequences present not only in viable cells but also in non-viable cells preventing their differentiation, such issues can be solved using reverse transcriptase PCR or qPCR techniques that also detect viable cells [12,17,43].

### 3.2. Reverse Transcriptase Polymerase Chain Reaction—RT-PCR

In a reaction using RT-PCR, the RNA molecule is transcribed to a complementary DNA molecule (cDNA). After this procedure, amplification is done using standard PCR. It is a method of high specificity, sensitivity and reliability [13,43]. The cDNA molecule formed from the initial RNA has been shown to have a higher level of purity compared to a DNA molecule withdrawn directly from the target. A standard DNA molecule contains impurities such as proteins that are not found in a cDNA molecule. Therefore, cDNA is more specific and more easily recognized by the primers [4]. Another advantage is that the technique allows the detection of replicating cells, with high sensitivity to differentiate living from dead bacteria. This is extremely important for determining the real risk of consuming ABF contaminated with living bacteria capable of causing diseases which are difficult to treat due to the high expression of AMR genes. RT-PCR also is used to study gene expression qualitatively and is essential for performing other molecular techniques such as qPCR for quantifying RNA levels and microarray for detecting multiple target gene expression [49,50]. RT-PCR is routinely used in experiments involving eukaryotic cells to differentiate exons from introns and can be used to diagnose genetic diseases and monitor antimicrobial drug therapy [50,51]. Conversely, the great disadvantage of this method is the high instability of the RNA molecule. For that reason, sample processing is the biggest challenge and requires qualified and prepared personnel, making analysis extensive and more expensive [53].

Researchers have used RT-PCR to develop a method for detecting resistance genes in meat. They were able to identify, in plasmids, genes for resistance to neomycin and puromycin [54].

The use of RT-PCR to detect AMR genes is not so widely described in the literature. However, most studies often use the technique to detect contamination from bacteria and viruses, as well as toxins produced by bacteria in the food production chain, and obtain interesting results [55,56].

### 3.3. PCR Combined with Restriction Fragment Length Polymorphism—PCR–RFLP

PCR–RFLP is based on DNA fragments obtained through digestion of amplified DNA by restriction enzymes (endonucleases). The fragments are made by recognizing unique nucleotide sequences, which can be read by RFLP to confirm the target sequence (e.g., genes that encode AMR) [57]. Because it is a complex technique that demands several steps, it is also disadvantageous due to the long time required to obtain results and the high cost of some restriction enzymes. Nevertheless, RFLP has proved to be efficient for the identification of resistance genes, an objective not achieved using other similar molecular techniques such as RAPD or pulsed-field gel electrophoresis [58].

Alonso et al. [59] identified mutations in the *gyrA* gene associated with AMR in *Campylobacter* with PCR–RFLP. All ciprofloxacin-resistant isolates (previously identified by the MIC technique) showed a point mutation at the Thr-86 position of the *gyrA* gene. In *Salmonella*, the PCR–RFLP technique was able to identify mutations in the quinolone resistance-determining region of the *gyrA* gene in strains resistant to nalidixic acid in poultry samples, showing that such point mutations may effectively contribute to AMR [60]. Overall, RFLP and all techniques based on PCR are very well suited as a complement to traditional techniques in studies focused on AMR gene detection. They identify genes responsible for transferring resistance to antimicrobials, as well as accurately identifying the species of the isolates studied.

### 3.4. Real-Time Polymerase Chain Reaction—qPCR

In qPCR, it is possible to detect and quantify the amplified portion of DNA during the whole amplification process [57]. This quantification is made possible by the inclusion of fluorescent probes to specific primers that emit detectable signals [43,61]. The addition of fluorescent probes such as molecular beacons to PCR amplifications makes possible real-time monitoring of amplification. Compared to the conventional PCR technique, qPCR has a shorter reaction time and it is possible to determine the relative and absolute number of microorganisms in the sample [57].

However, the cost of equipment and reagents is higher compared to standard PCR. In addition, the reaction can suffer inhibition by microorganisms from the environment or the food matrix that are not the target of the research [57].

Sometimes, only the presence of a resistance gene is not enough for a clinical outcome of AMR. For example, if the presence of β-lactamase genes in some clinical isolated bacteria does not correlate well with the effect of penicillin treatment, it is possible that presence of the resistance gene does not determine treatment failure because the level of gene expression may be too low [20]. In this context, the use of RT-qPCR is fundamental for quantifying the expression level of AMR genes.

As bacteriophages can package host genetic material, including genes that translate resistance to antimicrobials, researchers have used qPCR to research phage particles containing resistance genes in different types of meat. Genes such as *sul1* and those that determine β-lactamase resistance have been identified in meat samples [60]. Additionally, with the aid of qPCR, resistance genes have been investigated both in goat and sheep meat as well as in the slaughterhouse environment. Researchers confirmed the presence of *tetA* and *tetB* genes in several areas of the slaughterhouse as well as in meat products [62]. Using qPCR, it was possible for researchers to identify the presence of resistance genes in other types of meat product; *tetA* and *tetB* genes were found in these different samples [37].

### 3.5. Microarray

The microarray is another technique worth mentioning for investigation of genetic AMR in bacteria. This technology allows the study of gene expression by hybridization of oligonucleotide sequences that will purify and amplify specific molecules of RNA from the sample of interest. This allows its use for several purposes, mainly for identifying the function of certain genes [37,58]. The technique has long been used as the gold standard for the study of transcriptomes. Microarray use in the detection of AMR genes can be optimized by several hybridization processes and done simultaneously on the same substrate (e.g., glass, membrane or gel pad) that have many probes [37,63,64]. Another great advantage is that previous culture of bacteria is not necessary, as the DNA sample can be directly isolated to make the microarrays [47].

Microarray use has been otherwise replaced in recent years by new-generation sequencing techniques. One limitation of microarrays, for example, is the need for prior knowledge of the genomic regions to be studied. In addition, by investigating only predetermined target regions, one may end up missing important and critical information in the samples. Another major problem is the hybridization of similar sequences, complicating the reading and analysis of target genes [65].

Baumgartner et al. [66] used the microarray technique to identify resistance genes in ready-to-eat food. Resistance genes referring to methicillin (*mecA*), vancomycin (*vanB*), macrolide (*msr*), tobramycin (*aadD*), tetracycline (*tet*) and chloramphenicol (*cat*) were found. Vogt et al. [67] identified third-generation cephalosporin-resistant *Escherichia coli* in different samples of different types of meat. In addition, their results revealed genes conferring resistance to chloramphenicol (*cmlA1*-like), sulfonamides (*sul*), tetracycline (*tet*), and trimethoprim (*dfrA*). Morach et al. [68] also used the microarray technique, to search for pathogenic microorganisms as well as genes that decode enterotoxins. In general, they found low prevalence of *S. aureus*, and the genes decoding enterotoxins found were *sea*, *seb*, *sec*, *seh*, *sel* and *egc*. Kittler et al. [69] detected *S. aureus* resistant to methicillin in chicken meat samples from different farms along with the genes for λ-hemolysin (*lukF*, *lukS* and *hlgA*), leucocidin (*lukY* and *lukX*), and hemolysin (*hl*, *hla*, *hIIII* and *hlb*).

Clearly, even though the revolutionary aspects brought through the microarray technique to genetic AMR detection research, next-generation sequencing techniques have several advantages, overcoming the limitations of the microarray which is gradually becoming useless for AMR gene investigation over time.

### 3.6. Whole-Genome Sequencing and Metagenomics

The WGS DNA extracted from the tested samples is assembled by programs based on a De Bruijn graph (DBG), such as SPAdes, Velvet, ABySS and SOAPdenovo. The assemblies formed from small sequencing reads are called contigs, which can be annotated to search for resistance genes. The search for resistance genes is mostly done by methods (e.g., BLAST, USEARCH, DIAMOND) that consider the similarity of the contigs to the genes contained in reference databases, such as Resfinder, ARG-ANNOT, RGI, ARGs-OAP, RGI, ARGs-OAP (v2), ARIBA, PointFinder, NCBI-AMRFinder, SRST2, SEAR, ShortBRED, PATRIC, SSTAR, KmerResistance, GROOT and DeepArgs, as cited above. The choice of database depends on both the purpose of each study (i.e., resistance genes, virulence genes, proteins) and on the sequence confidence deposited in each database [70]. Since the recent improvement in the cost–benefit ratio of sequencing technologies, whole-genome sequencing (WGS) has become easily accessible and an effective tool in antibiotic resistance, a major threat to modern healthcare. WGS has already overcome numerous paradigms in this area, ranging from the development of novel antimicrobial drugs and diagnostic tests to real-time surveillance and elucidation of the factors that allow the emergence and persistence of resistance.

Multiple studies [32,43,71,72] have proved the value of WGS as a technique well suited to routine infection control and, for some pathogens, as a primary diagnostic tool to detect antibiotic resistance. In particular, Oniciuc et al. 2018 [73], compiled a review article summarizing the information currently available on the use of WGS and WMS for surveillance of AMR in foodborne pathogenic bacteria and food-related samples and discussed future needs that will have to be considered for the routine implementation of these next-generation sequencing methodologies with this aim. They identified that most WGS applications for surveillance of AMR genes in foods available in the literature arise from studies conducted in the last five years and are focused on high-priority foodborne pathogens, such as *Salmonella*, *Campylobacter* spp., Shiga toxin-producing *E. coli*, *Listeria monocytogenes* or *S. aureus*. Furthermore, the compiled studies have been aimed at discriminating resistant isolates coming from different sources, identifying AMR genes mechanisms or genetic determinants of resistance, defining and attributing infection sources in cases of food-related outbreaks caused by resistant microorganisms, or tracking the dissemination of AMR through the transfer of resistance genes. Conversely, WGS applications for surveillance of AMR also showed drawbacks. A very recent paper revealed causes of disagreement between the WGS genotype and experimentally determined AMR phenotype [74]. The study presented five instances of heteroresistance in *S. enterica* in conjunction with the corresponding failure of WGS to predict the phenotypic resistance due to a lack of genotypic resistance determinants. The WGS failures were attributed to the possible resistance being mediated by unstable genetic features such as temporary genes amplification. Even the best or most suitable bioinformatics software and sequencing technologies cannot avoid gaps in the genome during assembly obscuring resistance genes’ true presence in the final assembled genome. Furthermore, bioinformatics tools and databases would need to be adapted and continuously updated to understand the resistance mechanisms and characterize the possible AMR threat in food samples. Other challenges for using WGS in food resistance analysis are lack of standardization in the collection of samples and in the use of bioinformatics tools which directly interferes in the results, the difficulty in attributing the identified resistance genes to specific strains and the interferences due to the presence of genetic material of non-microbial origin is also a problem in many types of food samples. Even with so many challenges, the WGS technique has great potential in tracking resistance in food samples [72,73].

Metagenomics has also recently been used to detect and characterize foodborne pathogens and their genotypic resistance [13], and can characterize microbial communities without the need for culture [75,76]. In this approach, sequencing of the whole community’s genetic material is performed, and the advantages and disadvantages depend on the chosen sequencing database for the detection of resistance genes, i.e., SRST2, SEAR, ShortBRED, PATRIC, SSTAR, KmerResistance, GROOT, DeepArgs, Resfinder, ARG-ANNOT, RGI, ARGs-OAP (v2), ARIBA, PointFinder and NCBI-AMRFinder [13,70]. The advantages and disadvantages characteristic of these AMR gene databases were unraveled by Boolchandani et al. [70]. Metagenomic sequencing is efficient in evaluating the ecology of microorganisms and their changes during the processing of animal products (e.g., changes in temperature and available substrates that generate stress and cause the reorganization of microbial communities, affecting their persistence in the production chain) [61]. Additionally, it is possible to characterize the natural microbiota of animals in addition to discovering new genes and new microorganisms, including those that are not cultivable [77]. Along with new-generation sequencing, a large database of sequences from various environments is obtained [78]. Traditional or even gene amplification methods are limited compared to metagenomics because of some cultivation limitations and the need for prior knowledge of resistance genes to make primers for the amplification. In addition to being a technique that contributes to a complete understanding of ecological relations, metagenomics helps to expand knowledge about the studied microorganisms as well as the dynamics of their genes and possible resistomes [77]. Understanding microbial dynamics contributes to the biotechnological advances of new therapeutic discoveries, as well as helping to identify the contribution of these microorganisms to animal and human health [79].

Studies based on metagenomics have elucidated the effects of antimicrobial use in the breeding of production animals due to possible detection of the changes that occur in the microbial population [13]. Although it has the disadvantage of being an expensive technique, it is a reliable alternative for obtaining detailed analysis in studies involving bacterial resistomes [28]. In addition, because metagenomics is an open-approach molecular technique, it has some limitations such as the possibility of not providing deep sequencing of the genome of a species, especially if the samples come from complex communities such as those found in soils. Another challenge is encountered when the study focuses on contaminating DNA, but when it is associated with a host, the data obtained in the sequencing may come mostly from the host [80].

Auffret et al. [81] proposed that the intestinal microbiota of beef cattle can be a reservoir for pathogens and AMR, and this environment can be influenced by the animals’ diet. They found 204 genes associated with both resistance and pathogenicity, the most common antibiotic resistance genes being those against chloramphenicol and microcin, and demonstrated that diet can interfere in the presence of AMR genes. Singh et al. [82] used metagenomics to identify AMR and virulence genes in the ruminal microbiota of Indian buffaloes, using pyrosequencing technology to characterize the microbiological diversity of the buffalo microbiome. As a result, more than 6% of the analyzed sequences can be considered genes of resistance and virulence, this ratio being bigger than those found in ruminal cow or cecal bird microbiomes. Hence, this approach shows that it is an important tool for tracking AMR genes as well as genes related to the virulence of the microbiome. Moreover, such data can be used to create profiles of AMR genes, facilitating understanding of the ecology of microorganisms in their habitat.

Thomas et al. [83] studied the impacts on cattle intestinal microbiota generated by the use of antibiotics as additives. Using metagenomics, their results demonstrated that the use of additives does not cause apparent changes at the bacteria phylum level but decreases the amount of gram positive bacteria at the genus level. The number of *Ruminococcus*, *Erysipelotrichaceae* and *Lachnospiraceae* was reduced when analyzed in steers. However, no relationship was shown between the presence of AMR genes and the administration of antibiotics as feed additives in the studied animals. In contrast, Xiong et al. [84] undertook a comprehensive metagenomic analysis to identify both changes in the microbiota and variations in AMR genes in feces from broiler chicks treated with therapeutic doses of chlortetracycline. Even at therapeutic doses, the treatment resulted in an increase in tetracycline resistance genes. The effects of chlortetracycline on microbiota resistance are related to specific types of resistance genes and not to general resistance genes. The study contributed to improve treatment regimens, making them more effective during the treatment of resistant pathogens on farms. Ma et al. [71] analyzed resistance genes and shared resistance between pigs, chickens and humans in fecal samples using metagenomics. High levels of tetracycline, erythromycin and aminoglycoside resistance genes were found, in addition to multi-resistance genes. Their results demonstrate the possibility of identifying the hosts of these resistance genes besides demonstrating the sharing of the same resistome between different host. All these scientific studies discussed above are successful examples where metagenomics has opened new possibilities for a knowledge frontier breakthrough, making the access of AMR dynamics much more efficient and confident.

Raw chicken meat is a staple food with relatively high microbial loads. For this reason, there is a need to use microbiomes to inform multiple safety and food quality characteristics. In this context, Li et al. [85] evaluated the microbiome and resistome in samples of chicken breast from US retail establishments processed in the 3 main chicken production states. The evaluation was made through shotgun metagenomics on the microbiome’s specific constituents, such as AMR genes. A total of 8 samples of chicken breast products from 4 establishments processed by 5 brands for 7 months (from July 2017 to January 2018) were collected. Furthermore, the way the products were packaged, vacuum versus normal air-permeable packaging, were compared. As a result, it became apparent that the dominant bacteria in vacuum-packed products were more diverse, including genera such as *Aeromonas*, *Enterobacter*, *Lactococcus*. In comparison, the air-permeable samples were dominated by *Pseudomonas*. Another important result was the observation of resistance to 10 classes of antibiotics with 132 AMRs genes in chicken breast microbiomes. Most of the detected AMRs genes belonged to the classes of aminoglycosides and beta-lactams (53.8%). Furthermore, the abundance of AMRs genes in the vacuum-packed samples was 4.5 times greater than that of the air-permeable samples.

The eligibility of metagenomics for AMR research shows it to be the best option for a very large amount of bacterial assays. In studies focusing on the detection of resistant bacteria in organic pig rearing, for example, metagenomics was also found to be a suitable method for determining the presence of resistance genes in this breeding system. In this context, Kazimierczak et al. [86] analyzed 9000 bacterial chromosomal clones. They demonstrated that even in an organic system, genes known to generate resistance against antimicrobial drugs such as tetracyclines and doxycycline can be found within the bacterial population, the best explanation for it being the presence of mobile genetic elements. Additionally in a pig breeding system, Lamendella et al. [87] analyzed 637,722 sequencing reads to better understand microbial diversity and yet could not well understand the functional capacity of the intestinal microbiota of pigs. However, they proved that genes associated with AMR are homologous to genes related to carbohydrate metabolism and concluded that the pig’s intestinal microbiome can be shaped by management practices.

Due to its ability to sequence a whole microbiome, metagenomics is an effective tool in the study and discovery of new resistance genes. It is a current and very effective technique which should be more explored in studies involving the meat production chain.

## 4. Final Considerations

In this review, we highlight the principal features of traditional and genetic assays for the detection of AMR, their advantages and limitations, and discuss the general applications in the field and in basic research. Table 1 summarizes the most evident advantages and disadvantages of the most important methods for the detection of AMR in bacteria. It shows the importance, in some aspects, of the need for using combined methods for the best accuracy during the identification of resistance genes. It is necessary to use a wide variety of genetic assays to confirm resistance gene determinants and to support doubtful phenotypic results, as well as to provide a reliable scientific basis for the molecular surveillance of AMR bacteria and resistance determinants on a global scale. To supply the necessary confidence and accuracy in genetic AMR determinants, metagenomics is highlighted as a rather more complete method compared to other molecular methods, since it can achieve all information for a microbiome present in the studied sample. However, there is no better or worse technique in terms of AMR determination, and the best combination of the advantages offered by each method depends on the query to be answered. Overall, the conscious use of antimicrobials, on all possible occasions, is the best call to control the increase and spread of AMR in bacteria, especially for drugs that are used in the treatment of both animals and humans. With greater awareness of the risks of indiscriminate use of these antimicrobials, future problems in the treatment of pathogens can be mitigated.

## Figures and Tables

**Table 1:** Summary of the advantages and disadvantages of the most common traditional and molecular methods for the identification of microorganisms and antimicrobial resistance genes.

Method	Advantages	Disadvantages	References
Traditional	Low cost, high sensitivity, gold standard for microorganism identification	Execution time, viable non-cultivable strains, low specificity	[14,25,43,88]
Conventional PCR	Amplification of viable non-cultivable microorganism genes	Does not detect cell viability	[12,17,43]
Multiplex PCR	Amplification of different types of genes at the same time	Primers with similar annealing temperature	[26,43,89]
RT-PCR	Detection of live cells, cDNA molecule has high purity, high specificity	Instability of the RNA molecule	[27,43]
PCR–RFLP	Cost, easy to design, doesn’t need expensive materials, easy to accomplish	Some enzymes can be expensive, delay in obtaining results	[31,90]
qPCR	Shorter reaction time, determines relative and absolute number of microorganisms	High cost, interference by environmental microorganisms	[13,43,57]
Microarray	Does not need previous culture of the studied bacteria, detection of several resistance genes simultaneously on the same substrate	Need previous knowledge about genomic regions to be studied, region to be studied is previously determined causing loss of additional information	[37,47,64,65,91,92]
Metagenomics	Detection of whole microbiome, no previous culture required, discovery of non-cultivable microorganisms. New genes and microorganisms	Need prior knowledge in bioinformatics, high cost, challenge of achieving deep sequencing of more complex microbiomes	[28,75,80,93]

## Data Availability

Not applicable.
